# A model based on equations of kinetics to study nitrogen dioxide behavior within a plasma discharge reactor

**DOI:** 10.1186/s40201-015-0228-5

**Published:** 2015-10-08

**Authors:** Mehdi Abedi-Varaki, Alireza Ganjovi, Fahimeh Shojaei, Zahra Hassani

**Affiliations:** Department of Plasma Engineering, Graduate University of Advanced Technology, Kerman, Iran; Photonics Research Institute, Institute of Science and High Technology and Environmental Sciences, Graduate University of Advanced Technology, Kerman, Iran; Department of New Materials, Institute of Science and High Technology and Environmental Sciences, Graduate University of Advanced Technology, Kerman, Iran

**Keywords:** Non-thermal plasma, Dielectric barrier discharge, Plasma reactor

## Abstract

In this work, a zero-dimensional kinetics model is used to study the temporal behavior of different species such as charged particles, radicals and excited states inside a Dielectric Barrier Discharge plasma reactor. It is shown that, the reactor significantly reduces the concentration of nitrogen monoxide as an environmental pollutant. After a drastic increase, a decrease in the concentration of the NO_2_ molecules inside the reactor is seen. Nitrogen monoxide molecules with a very low concentration are produced inside the reactor and its quick conversion to other products is proved. The obtained results are compared with the existing experimental and simulation findings, whenever possible.

## Introduction

Nowadays, high voltage electrical discharge plasmas and their applications in many practical fields such as biology, chemistry, electrochemistry and environmental programs are being widely studied. The plasma discharge technique can be used for reforming of the poisonous pollutants, such as SO_x_, CO_x_ and NO_x_. Generally, the Dielectric Barrier Discharge (DBD) plasma reactor is supposed to be cylindrical and can be installed on the factory chimneys. In comparison with the other methods such as selective catalytic reduction method, it has shown better efficiency and fewer side problems [1].

Nitrogen dioxide (NO_2_) is a yellowish brown or reddish brown and of course, an invisible gas and is converted to suspended nitrate during the complex processes in the atmosphere. Its environmental concentration due to activities of gas power plants, industrial factories and diesel vehicles increases. Moreover, the generation of this pollutant gas, owing to the high temperature fuel combustion, is higher. The mixture of NO_2_ gas with the moist air may produce citric acid leading to the materials corrosion [2].

According to the existing reports, the extra emission of toxic pollutants from chimneys of factories will affect the health of workers, since the working environment has been severely compromised. However, it should be pointed out that these gases act as a leading factor and have an effective contribution in the acidic rain and formation of dense smog and smoke and chemical fumes. Thus, the result is an extremely harmful effect on the human health conditions, especially for the worker's environment and residential areas at the factories surrounding [3].

So far, some studies on the generated plasma discharges by DBD as a composition with different catalysts have been performed. These studies are based on the simulation of reactors for the reduction of NO_x_ in the diesel engine exhaust gases. In these systems, the energetic electrons and free radicals with the introduced catalysts get generated as plasma to reduce the NO_x_ concentration inside the reactor [4–10].

Mei-Xiang et al. studied the simultaneous removal of NO_x_ and dust from diesel exhaust based on the mixture of metal catalyst and plasma discharge [11]. The reduction effect of enough amount of oxygen on the decomposition of NO_x_ and small particles in the atmospheric pressure was observed. The creation of CO_2_ and N_2_ inside the reactor regardless of N_2_ temperature was reported. According to their results, the temperature decreases for combustion and the reduction of the conversion efficiency of NO_x_ to NO increases during the plasma formation. It shows that, the catalytic activity can be improved via the plasma formation process.

Yamamoto et al. presented a chemical reactor conjugated with plasma discharge as a strategy for decomposition of NO_x_ pollutants [12]. In their system, the plasma nitrogen monoxide was converted to nitrogen dioxide and, consequently, the conversion of nitrogen dioxide to nitrogen was occurred during the reduction mechanisms. Moreover, they studied three plasma reactor models and, the decomposition of almost 100 % of nitrogen dioxide was observed.

Dorai et al. studied the interactions between soot particles and NO_x_ molecules in the exhaust of diesel engine during electrical discharge process using a zero-dimensional chemical model [13]. They computationally investigated the effects of soot particle on the chemical plasma in the DBD reactor.

Fujii et al. considered a DBD reactor with film coating on the ground electrode to eliminate NO_x_, CO_x_, SO_x_ and soot particles [14]. They concluded that the NO_x_ reduction is approximately limited to 70 %.

Wang et al. used the DBD method for the formation of non-thermal plasma and reforming of carbon dioxide inside the plasma reactor. They separately studied the effects of catalytic and non-thermal plasma on the reforming of carbon dioxide. They found that, the temporal reduction of CO_2_ concentration using DBD reactor is much stronger than the other methods [15].

In this work, the temporal behavior of different species and their reaction rates are studied using a zero-dimensional model based on equations of kinetics. The considered model is able to describe the behavior of these species such as charged particles, radicals, excited states.

## Simulation model

**Fig. 1:**
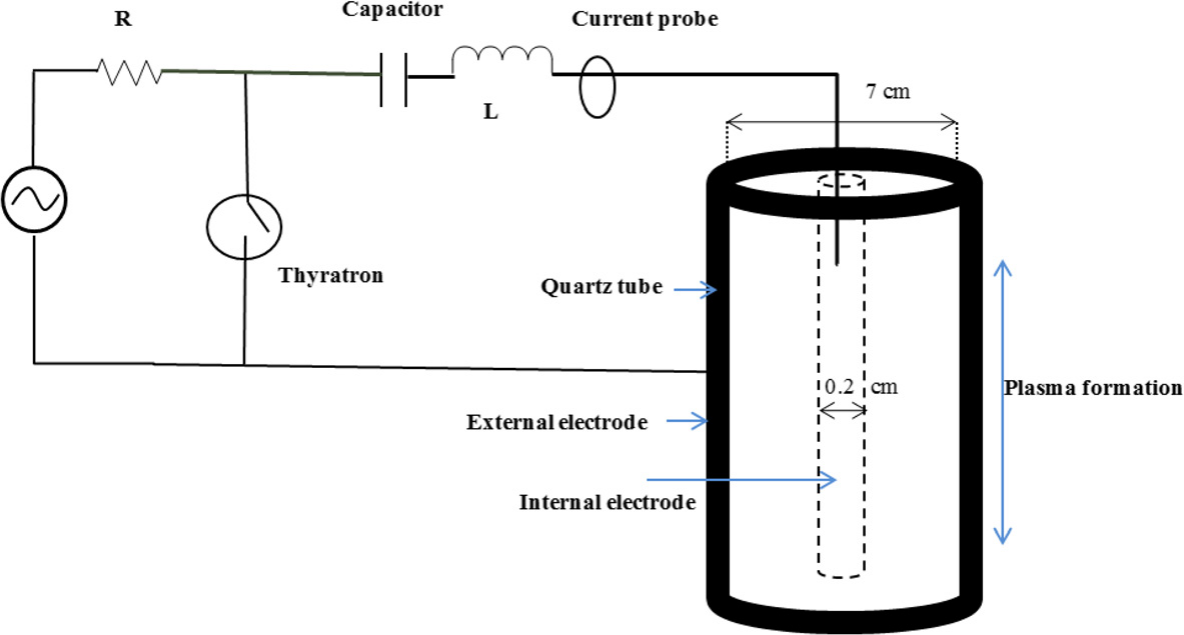
Schematic of the DBD plasma reactor

In a comprehensive and complete model to study the conversion of the nitrogen dioxide molecules and their temporal behavior within the plasma discharge reactor, the following phenomena should be accounted for: (a) the temporal variations of the different charged species and their corresponding reaction rates, (b) the ionization coefficients inside the reactor. Because of the strong interaction of these phenomena inside the DBD reactor, complete simulation of the different formed species inside the plasma reactor which encompasses these phenomena together, is a very challenging task. One can attack this plasma reactor modeling problem based on the equations of kinetics. As shown in Fig. 1, the reactor is considered to be cylindrical with the inner and outer electrodes and the density of background nitrogen dioxide gas is assumed to be constant. The inner electrode is connected to the High Voltage Alternating Circuit (HVAC).

The different parts of this plasma reactor are described in the following: The quartz tube is chosen as the dielectric material owing to its high melting point and transparency. As seen in Fig. 1, this tube has a thickness of 0.05 cm, inner diameter of 0.2 cm and outer diameter is 7.0 cm. Internal and external electrodes are both assumed to be stainless steel. The diameter of inner electrode is considered to be equal 0.2 cm. The inner electrode and outer electrode are connected to the high voltage (HVAC).

Furthermore, while the gas movement along the chimneys axis, application of HVAC along the radius and the consequent appearance of a radial electric field causes the occurrence of electrical glow discharge resulting in the formation of non-thermal plasma and changes in nitrogen dioxide reactions. These changes along with constant reaction rates are considered in the considered model which is based on equations of kinetics. The rate constant for all reactions at temperature 300 K and atmospheric pressure are presented in Table 1. During the electric discharge process, different reactions will happen and different particles are produced in the plasma reactor volume.

**Table 1:** Nitrogen dioxide gas reactions in the presence of non-thermal plasma

Reactions	Rate constant (*m* ^3^. *s* ^− 1^)
${O}^{-}+ NO\overset{k_1}{\to }N{O}_2+e$	*k*_1_ = 8.3 × 10^− 40^
${O}^{-}+{N}_2\overset{k_2}{\to }{N}_2O+e$	*k*_2_ = 1.6 × 10^− 41^
$N{O}_2+e\overset{k_3}{\to }N{O}_2^{-}$	*k*_3_ = 4 × 10^− 17^
${O}_2+e\overset{k_4}{\to }{O}^{-}+O$	*k*_4_ = 5.27 × 10^− 17^
$N{O}_2+O\overset{k_5}{\to } NO+{O}_2$	*k*_5_ = 9.6 × 10^− 18^
${N}_2^{+}+N{O}_2^{-}\overset{k_6}{\to }N{O}_2+{N}_2$	*k*_6_ = 3 × 10^− 12^
${N}_2+e\overset{k_7}{\to }{N}_2^{+}+2e$	*k*_7_ = 6.66 × 10^− 17^
${O}_2+e\overset{k_8}{\to }{O}_2^{+}+2e$	*k*_8_ = 1.96 × 10^− 16^
${O}_2^{+}+N{O}_2^{-}\overset{k_9}{\to }{O}_2+N{O}_2$	*k*_9_ = 2 × 10^− 12^

The considered model describes discharge process between outer and inner cylindrical electrodes in the reactor volume. The general balancing equations for each species, i.e., electrons, neutrals, ions and radicals can be written as follows [16]:

1 $$ \frac{\partial nj}{\partial t}-{D}_a{\nabla}^2{n}_j=Rj $$

Where D_a_ is the am-bipolar diffusion coefficient and R_j_ is the reaction rate for the j^th^ species. Moreover, as mentioned before, the inner electrode is connected to HVAC power source in order to apply the electric field along the radial direction in the reactor volume.

In this work, the essential reactions are taken into account and the creation of eight species during discharge process is considered. Atomic oxygen is a vital particle in the dissociation of nitrogen dioxide inside the plasma reactor. According to Table 1, the production of NO, NO^−^_2_ and O_2_ is based on dissociation of NO_2_ in the reactor. Moreover, the formation of NO_2_ also occurs along three possible routes. In the first route, electron is produced due to electrical discharge and in the second route, the nitrogen atom is generated. In the third route, atomic oxygen is created via dissociation of NO^−^_2_. Distinction between these three routes is important, since the nitrogen monoxide participates in the nitrogen dioxide production.

Since, except electrons, the mass of all the formed particles (Table 1) are comparable to that of neutrals, they can exchange energy efficiently in collisions with neutrals. Therefore, their temperatures are assumed to be constant and equal to the neutral gas temperature. Thus, the diffusion phenomenon and energy equation for these heavy particles is not needed. Then, based on the chemical reactions in Table 1 and Eq. (1), the continuity equations for electron and the other charged species are as follows:

2 $$ \frac{\partial {n}_e}{\partial t}-{D}_{ae}{\nabla}^2{n}_e={R}_e $$

3 $$ \frac{\partial {n}_{N{O}_2}}{\partial t}={R}_{N{O}_2} $$

4 $$ \frac{\partial {n}_{O^{-}}}{\partial t}={R}_{O^{-}} $$

5 $$ \frac{\partial {n}_{NO}}{\partial t}={R}_{NO} $$

6 $$ \frac{\partial {n}_{N_2}}{\partial t}={R}_{N_2} $$

7 $$ \frac{\partial {n}_{O_2}}{\partial t}={R}_{O_2} $$

8 $$ \frac{\partial {n}_O}{\partial t}={R}_O $$

9 $$ \frac{\partial {n}_{N_2^{+}}}{\partial t}={R}_{N_2^{+}} $$

10 $$ \frac{\partial {n}_{O_2^{+}}}{\partial t}={R}_{O_2^{+}} $$

11 $$ \frac{\partial {n}_{N{O}_2^{-}}}{\partial t}={R}_{N{O}_2^{-}} $$

Where, we have:

12 $$ {R}_{\alpha }={K}_i{n}_{\alpha }{n}_{\beta } $$

To study the temporal behavior of the electrons in the plasma discharge reactor, the energy relaxation lengths are important, since they contain the information of energy gain process from the electric field and energy loss process owing to inelastic collisions inside the reactor. Spatial energy relaxation depends on the ratio between the mean free path for energy loss and the relevant length scales in the glow discharge plasmas. Generally, the kinetic energy for electron energy distribution function n(ε, t) is written in the following convenient form [16]:

13 $$ \frac{\partial n}{\partial t}=-\frac{\partial J}{\partial t}+{Q}^{*}+{Q}_i-{v}_a\left(\varepsilon \right)n-{v}_d\left(\varepsilon \right)n $$

14 $$ \begin{array}{l}J=-A\varepsilon \frac{\partial n}{\partial \varepsilon }+\frac{A}{2}n+n{V}_{el},\\ {}A=\frac{2}{3}\frac{e^2{E}^2{\nu}_m}{\omega^2+{\nu}_m^2},\kern1.8em {V}_{el}=-\frac{2m}{M}\varepsilon {\nu}_m\end{array} $$

The flux J along the energy axis reflects energy gains from the field and elastic losses. The term Q* describes the excitation of atoms, Q_i_ represents the ionization process and the attachment and diffusion losses are given by ν_a_(ε)n and ν_d_(ε)n, respectively [16].

On the other hand, electrons are not able to get very high energies inside the plasma reactor. This is due to the inevitable excitation and ionization events at energies above the corresponding potential. At energies ε → 0, the electron energy distribution function falls off very rapidly and the energy flux vanishes, i.e., J(∞) = 0. The particle sources in Eqs. (13) and (14) are distributed along the ε-axis. Moreover, there are no electron sources with zero energy and the negative kinetic energy is impossible and thus, it results in J(0) = 0. If we turn to an analogy with the one-dimensional diffusion of particles in the ordinary space, x ≡ ε, the situation is found to correspond to an impenetrable and non-emitting wall at x = 0. Consequently, the Eqs. (13) and (14) can be integrated over the entire spectrum from zero to infinity. The integral of Q* vanishes automatically, as the excitation process do not change the number of electrons. Integration in ε for Q_i_ yields -ν_i_n_e_, where ν_i_ is the ionization frequency averaged over the spectrum. The next term gives 2ν_i_n_e_; this is readily verified in the order of integration in the double integral [16]. Finally, the equation of kinetics for the electron density (Eq. 13) can be written as follows:

15 $$ \frac{d{n}_e}{dt}={\operatorname{R}}_e+\alpha \left|{V}_d\right|{n}_e $$

Where V_d_ and α are the drift velocity and primary ionization coefficient. Moreover, Eqs. (2)-(11) can be can be rewritten as follows:

16 $$ \begin{array}{l}\frac{d{n}_{N{O}_2}}{dt}={k}_1{n}_{O^{-}}{n}_{NO}-{k}_3{n}_{N{O}_2}{n}_e-{k}_5{n}_{N{O}_2}{n}_O\\ {}+{k}_6{n}_{N{O}_2^{-}}{n}_{N_2^{+}}+{k}_9{n}_{O_2^{+}}{n}_{N{O}_2^{-}}\end{array} $$

17 $$ \begin{array}{l}\frac{d{n}_e}{dt}={k}_1{n}_{O^{-}}{n}_{NO}+{k}_2{n}_{O^{-}}{n}_{N_2}-{k}_3{n}_{N{O}_2}{n}_e-{k}_4{n}_{O_2}{n}_e\\ {}+{k}_7{n}_{N_2}{n}_e+{k}_8{n}_{O_2}{n}_e+\alpha \left|{V}_d\right|{n}_e\end{array} $$

18 $$ \frac{d{n}_{O^{-}}}{dt}=-{k}_1{n}_{O^{-}}{n}_{NO}-{k}_2{n}_{O^{-}}{n}_{N_2}+{k}_4{n}_e{n}_{O_2} $$

19 $$ \frac{d{n}_{NO}}{dt}=-{k}_1{n}_{O^{-}}{n}_{NO}+{k}_5{n}_{N{O}_2}{n}_O $$

20 $$ \frac{d{n}_{N_2}}{dt}=-{k}_2{n}_{O^{-}}{n}_{N_2}+{k}_6{n}_{N_2^{+}}{n}_{N{O}_2^{-}}-{k}_7{n}_{N_2}{n}_e $$

21 $$ \frac{d{n}_{O_2}}{dt}=-{k}_4{n}_{O_2}{n}_e+{k}_5{n}_{N{O}_2}{n}_O-{k}_8{n}_{O_2}{n}_e $$

22 $$ \frac{d{n}_O}{dt}={k}_4{n}_{O_2}{n}_e-{k}_5{n}_{N{O}_2}{n}_O $$

23 $$ \frac{d{n}_{N_2^{+}}}{dt}=-{k}_6{n}_{N_2^{+}}{n}_{N{O}_2^{-}}+{k}_7{n}_{N_2}{n}_e $$

24 $$ \frac{d{n}_{O_2^{+}}}{dt}={k}_8{n}_e{n}_{O_2}-{k}_9{n}_{O_2^{+}}{n}_{N{O}_2^{-}} $$

25 $$ \frac{d{n}_{N{O}_2^{-}}}{dt}={k}_3{n}_e{n}_{N{O}_2}-{k}_6{n}_{N{O}_2^{-}}{n}_{N_2^{+}}-{k}_9{n}_{O_2^{+}}{n}_{N{O}_2^{-}} $$

As shown in Fig. 1, the electrodes are connected to frequency power source in order to change the radial electric field vector very quickly. Generally, the applied potential is written as follow [17]:

26 $$ V\left(z=L\right)=Vrf \sin \left(2\pi t\nu rf\right) $$

Where V_rf_ is the amplitude and set equal to 50 kV and υ_rf_ is power source frequency is set to 50Hz. The effects of the charged particles which are produced in the plasma reactor volume are neglected here.

Application of the external electric field between outer and inner electrode results in the ionization inside the reactors and formation of plasma discharge. Therefore, the Townsend's first coefficient or primary ionization coefficient (α (E/p)) and drift velocity (V_d_) of electrons in Eq. (15) are defined as follows [18, 19]:

27 $$ \alpha /p=A \exp \left(\frac{Bp}{E}\right) $$

28 $$ {V}_d={\mu}_eE $$

Where A and B are constant quantities, μ_e_ is electron mobility, L is the mean free path and V_i_ is ionization potential that are presented in the Table 2 [16, 18].

**Table 2:** Simulation data

Symbol		Value
Mobility, μ_e_, m^2^/Vs	For electron	0.144
A, constant quantity		1/L
B, constant quantity		V_i_/L
Temperature, T, K		300
Power source frequency, υ_rf_, Hz		50
Potential amplitude, KV		50
Primary density, m^−3^	For all species	10^8^
Primary density, m^−3^	For electron	10^7^
Time, s		10^−7^
Time-steps, s		10^−9^

## Simulation results

**Fig. 2:**
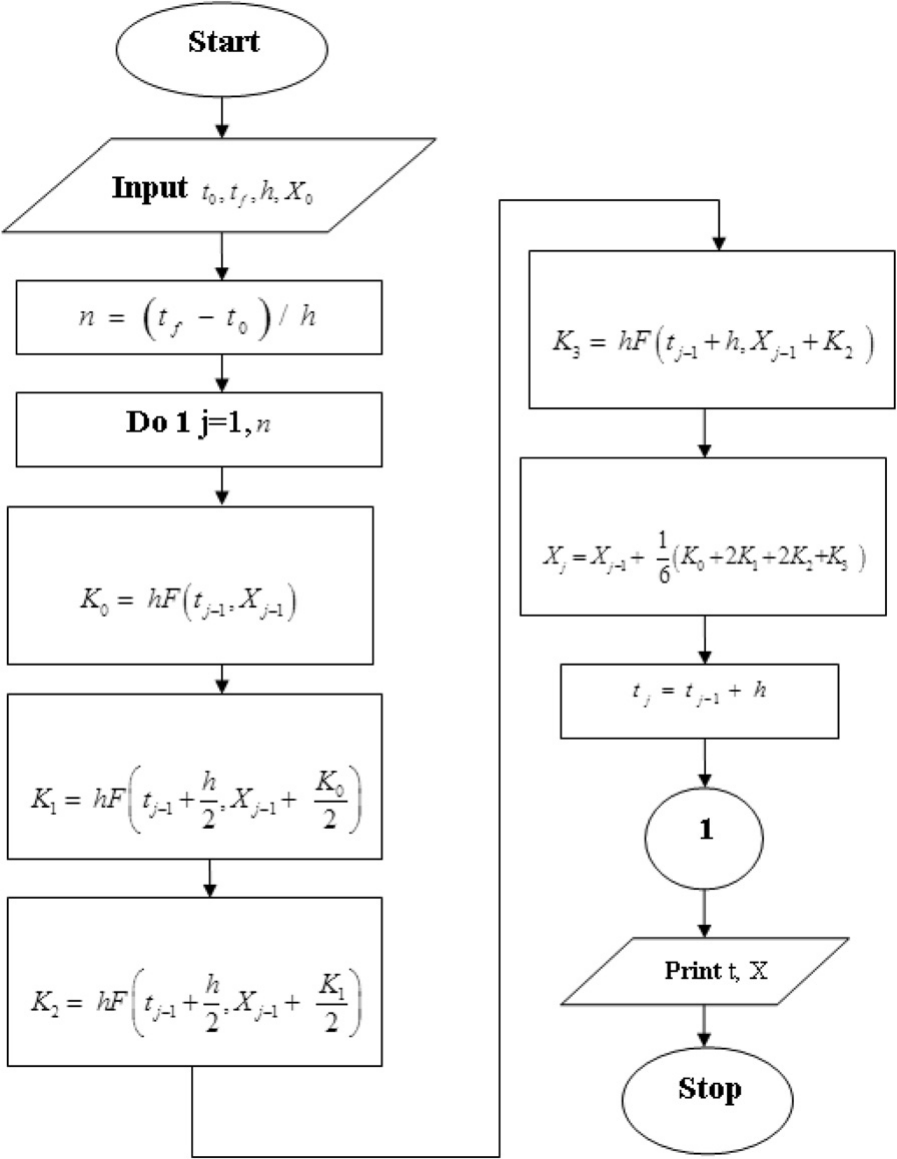
Flow-diagram of the mathematical modeling

In this section, the results obtained by solution of equations (15)-(25) to describe the temporal behavior of different species and their reaction rates are presented. The ODE45 routine from the Matlab software with the time-step of Δt = 10^−9^s is used to integrate these equations. The flow-diagram of steps in the mathematical modeling is shown in Fig. 2. It must be noted that, $X=\left\{{n}_e,\;{n}_{N{O}_2},\;{n}_{O_2},\;{n}_{O^{-}},\;{n}_{NO},\;{n}_{N_2},\;{n}_O,\;{n}_{N_2^{+}},\;{n}_{O_2}^{+},\;{n}_{N{O}_2^{-}}\right\}$ and t_0_, t_f_ and h are the initial and final moments and time step, respectively. Moreover, the simulation parameters used in this work are presented in the Table 2.

**Fig. 3:**
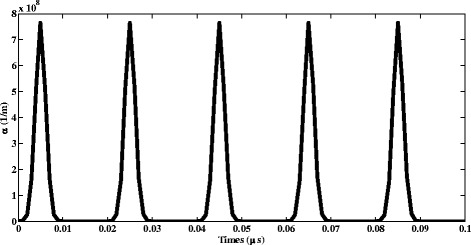
Temporal variation of primary ionization coefficient (α)

The primary ionization coefficient (α) is defined as the number of ions produced per unit path by a single electron traversing a gaseous medium between the electrodes with different polarities. This coefficient is fundamentally important in all the discharge processes to describe the electronic gain in the gaseous ionization media. The temporal variation of the primary ionization coefficient is presented in Fig. 3. Owing to the alternating variations in the applied electric field along the radial direction inside the plasma reactor, α varies alternatively.

**Fig. 4:**
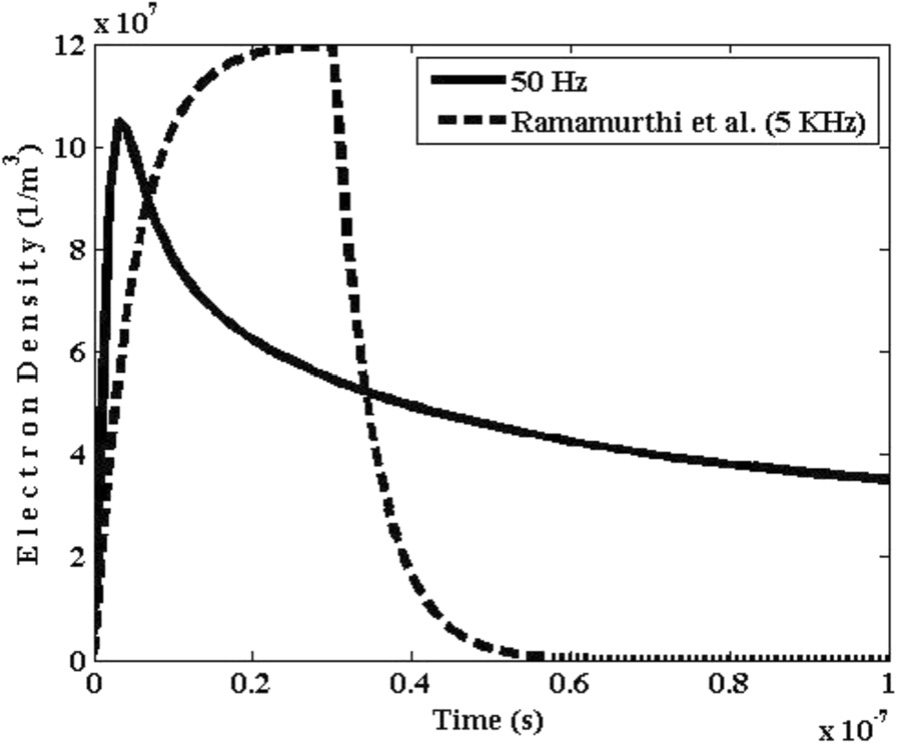
Temporal variation of number density of electrons

Figure 4 shows the evolution with time of the number density of electrons within the plasma reactor. It may be seen that, when applying the external electric field along radial direction between outer and inner electrodes, due to the ionization of gas and plasma formation, the density of electrons increases. It peaks at the moment of 0.05 s. Afterwards, the electrons growth owing to their attachment to NO_2_ and O_2_ (Table 1) will decrease. In fact, the formed plasma breaks the bonds between the different spices and causes the reforming gas. In this system, the formation of plasma discharge occurs via the ionization of NO_2_ inside reactor and it is followed by reforming of NO_2_ molecules. Moreover, as depicted in Fig. 4, the observed trend in the temporal variations of electrons density is in agreement with the findings by Ramamurthi et al. [20, 21].

**Fig. 5:**
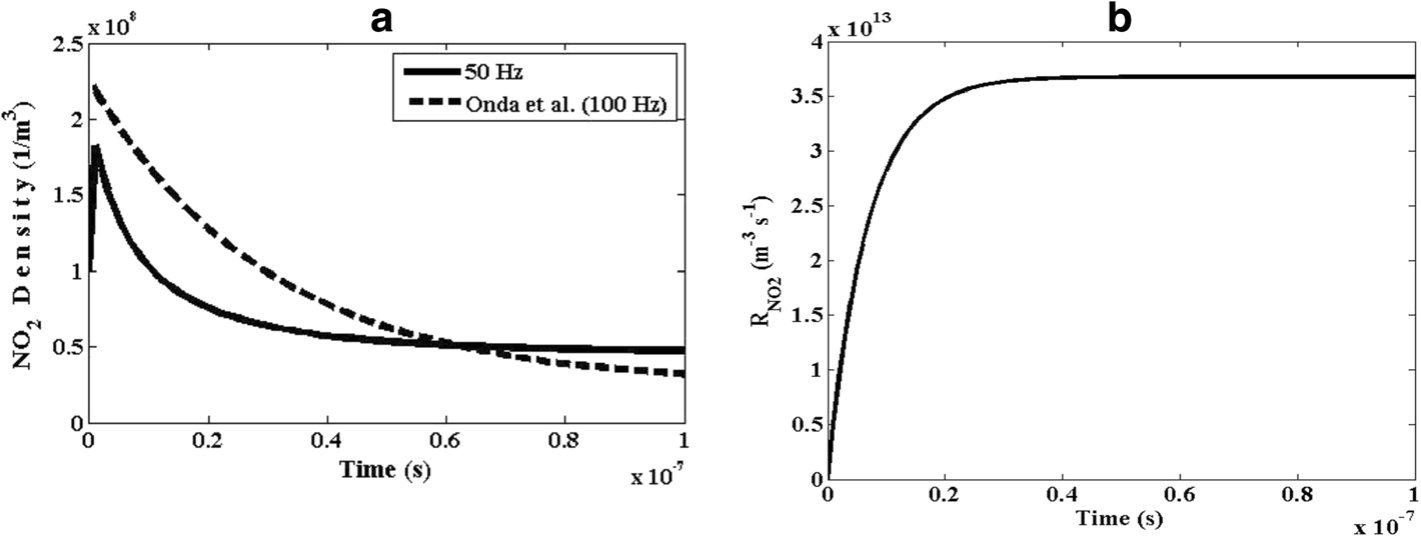
Temporal variation of **a** number density of NO_2_ and **b** the reaction rate of NO_2_

Figure 5a shows the temporal evolution of the number density of NO_2_ within the plasma reactor. According to Fig. 5, at t = 0, the initial number density of nitrogen dioxide is assumed to be 10^8^ m^−3^. As can be seen, after application of external electric field and discharge initiation inside the reactor, the number density of NO_2_ molecules, after a drastic increasing, decreases over time. Finally, it almost vanishes at the moment of t = 0.9 μs. This phenomenon confirms the ability of the reactor to remove NO_2_ pollutants. As shown in Fig. 5a, the observed trend of this finding is in agreement with the reported results by Onda et al. [22].

The reaction rate (R_j_ or R_reac_) is the speed of the chemical reactions and, generally, depends on rate constant (K), number density of particles, temperature and pressure in the gaseous media. The low reaction rate means that the molecules combine at a slower speed of than that of a reaction with a high rate. In this work, the gas temperature and pressure values are kept constant. Moreover, this reaction rate depends on the type of the combining molecules. The slower reaction rate is a direct result of the lower concentrations of an essential element or compound. To characterize the rate of a chemical reaction, having the rate of variations in the concentrations of the reactants and products might be useful. Figure 5b shows temporal variations of reaction rate of the nitrogen dioxide, R_NO2_. As can be seen, the reaction rate decreases and then, gets some saturated value within the plasma reactor. Since the number density of NO_2_ increases with time, the temporal variations of the chemical reaction speed for NO_2_ becomes faster.

**Fig. 6:**
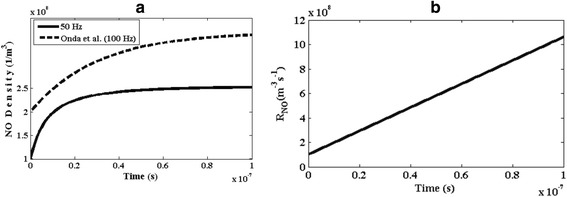
Temporal variation of **a** number density of NO and, **b** the reaction rate of NO

Nitrogen monoxide (NO) gas is a poisonous gas and has an effective role in the formation of acidic rain. But, it is an unstable composition and will be quickly converted to other species such as nitrogen dioxide. As seen in the Table 1, and is shown in Fig. 6a, the production of NO molecules occurs inside the plasma reactor. But, its concentration is very low and can be neglected. Moreover, the findings by Onda et. al. for NO density inside the reactor are depicted on the Fig. 6a [22]. Figure 6b shows the temporal variation of reaction rate (R_NO_) for nitrogen monoxide molecules. As seen, the speed of its chemical reaction is high which is similar to the other species. So, interestingly, this poisonous gas will be quickly removed using the DBD plasma reactor.

**Fig. 7:**
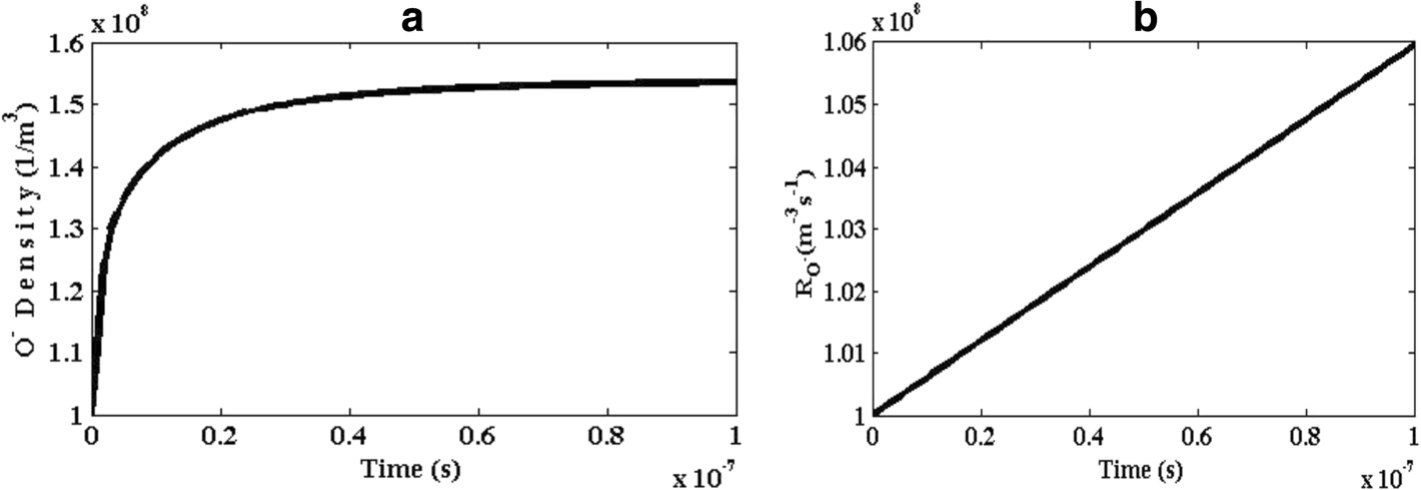
Temporal variation of **a** number density of O^−^ and, **b** the reaction rate of O^−^

The temporal variation of the number density of O^−^ ions within the plasma reactor is plotted in Fig. 7a. It can be seen that, from the moment of t = 0.04 μs onwards, the number density of O^−^ species gets saturated. It is clear that, not only this rector is able to remove NO_2_ pollutant agents from factories chimneys, but also it produces the harmless species such as is O^−^ ions. On the other hand, as seen in Fig. 7b, the reaction rate of the O^−^ increases. The increasing of the reaction rates of the O^−^ proves the instability and low period of their residing in the reactor. Moreover, oxygen ion (O^−^) is an unstable species and is quickly converted to the other species. Thus, they are not only harmless but also useful to the environment and play a significant role in the reduction of air pollution. On the other hand, they do not have enough time to have damaging collisions with reactor walls.

**Fig. 8:**
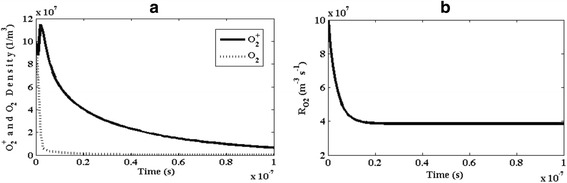
Temporal variation of **a** number density of O^+^_2_ and O_2_ and **b** the reaction rate of O_2_

Figure 8a shows the temporal variation of O_2_ and O^+^_2_ species. Positive ion of oxygen (O^+^_2_) is a harmless species which is produced in the electrical discharge process inside the plasma reactor. Since the concentration of oxygen atom reduces during the reaction, it decreases. Initially, because of the higher rate constant (2 × 10^−12^) of the positive ions of oxygen (O^+^_2_) in recombination with NO_2_^−^, its concentration reduces. This reduction is owing to the production of new ones based on the electron attachment. Latter on, from the moment of t = 0.05 μs, again its concentration, due to recombination with NO_2_^−^, decreases. Moreover, Fig. 8b shows the temporal variations of reaction rate of atomic oxygen (R_O2_). Since this reaction rate reduces, the temporal variation of the chemical reaction speed for O_2_ becomes slower.

**Fig. 9:**
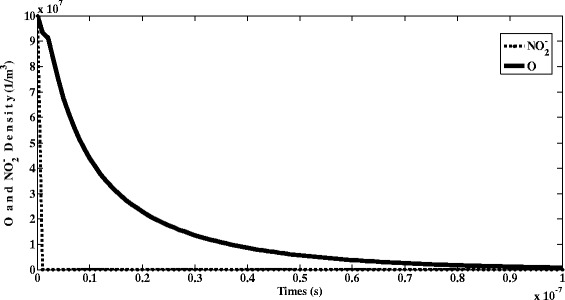
Temporal variation of number density of O and NO^−^_2_

Figure 9 represents the temporal variation of number density of atomic oxygen (O) and NO^−^_2_. It should be noted that O species is neutral and harmless, and its concentration decreases and does not affects the environmental pollution. As seen, NO^−^_2_ density decreases versus time. However, this species is unstable and is rapidly converted into the other species.

## Discussions

In this work, the temporal behavior of different formed species such as charged particles, radicals, excited states inside a DBD reactor and their reaction rates are studied using a zero-dimensional model based on equations of kinetics. It was found that, the considered model is able to describe the discharge process between outer and inner cylindrical electrodes in the reactor volume. As essential reactions, the creation of eight different species during discharge process inside the plasma reactor is taken into the account. Since, all the produced massive species exchange energy with neutrals, their temperatures are assumed to be equal to the neutrals. Moreover, the diffusion coefficients and energy equation is not considered for the formed heavy particle inside the reactor. The energy relaxation lengths contain the information for energy gain process from the electric field and energy loss process owing to the inelastic collisions inside the reactor.

As a result of alternating variations of the applied electric field inside the plasma reactor, alternating variations of the primary ionization coefficient (α) was observed. It peaks at 7.8 × 10^8^ m^−1^ at each every cycle. The number density of electrons within the plasma reactor peaks at the moment of t = 0.05 μs. It decreases owing to their attachment to NO_2_ and O_2_. The role DBD reactor in the significant reduction of the concentration of NO and NO_2_ inside the reactor was clear. Till the moment of t = 10 ns, the number density of NO_2_ molecule increases and, followed by a decrease. The saturation of number density of O^−^ was occurred from the moment of t = 0.04 μs onwards. Positive ion of oxygen (O^+^_2_) was seen to decrease as a result of reduction in the concentration of molecular oxygen. Moreover, as the reaction rate for this species reduces, the temporal variation of the chemical reaction speed for O_2_ becomes slower. Finally, it was seen that, NO^−^_2_ density decreases versus time.

## Conclusions

A zero-dimensional model based on equations of kinetics was successfully applied to study the temporal variations of the number density and the reaction rate of the nitrogen monoxide molecules and other produced species within the DBD plasma reactor. As was seen, the reactor has a significant reduction effect on the concentration of nitrogen monoxide as an environmental pollutant. It was observed that, by initiation of electrical discharge inside the reactor, the concentration of the NO_2_ molecules, after a drastic increase, decreases. Moreover, its chemical reaction speed becomes faster. The production of NO molecules with a very low density confirms that, this harmful gas is unstable and will be quickly removed using this plasma reactor.
